# Intradermal Cell Culture Rabies Vaccine: A Cost Effective Option in Antirabies Treatment

**DOI:** 10.4103/0970-0218.69287

**Published:** 2010-07

**Authors:** Asma Rahim, Kumaresan Kuppuswamy, Bina Thomas, Lucy Raphael

**Affiliations:** Department of Community Medicine, Calicut Medical College, Kerala, India. E-mail: rahim.asma@gmail.com

Sir,

In India, previously nervous tissue vaccines (NTV) were the mostly used vaccines for antirabies treatment, now this being replaced by modern, safe and effective cell culture vaccines (CCVs) due to NTV’s inherent neuroparalytic side-effects. But high cost and limited availability are the limiting factors for the wider use of CCVs.(1) Following WHO recommendations, results of clinical trials and international experience, Drug Controller General of India (DCGI) approved the use of safe, efficacious and feasible ID route of administration of CCVs from February 2006.(1) In the context of recent introduction of the ID regimen in Kerala in 2009, we undertook a retrospective analysis of case records to calculate the cost benefits of ID regimen, if it had been implemented in this tertiary hospital set-up for the past three years (2006-2008). Our main objectives were to assess the utilization of antirabies cell culture vaccines IM route for a period of three years in the preventive clinics, to find out the total cost of ARV used in this period and to compare the cost of IM regimen with ID regimen in terms of cost benefits.

We did a retrospective analysis of case records of a three-year period (2006-2008). All cases who have been treated with intramuscular ARV (both partial and complete) for a period of three years (2006-2008) in the preventive clinic of Calicut Medical College were included in the study. It was noted that during this span, purified vero cell vaccine (PVCV) supplied by the hospital was used which cost about Rs.280/vial in the market. The cost of ARV for three years was calculated and compared with intradermal regimen (modified thai schedule). The benefit in terms of expenditure to the Government was calculated if ID regimen had been used in all these cases.

Updated Thai regimen involves injection of 0.1 ml of reconstituted vaccine per ID site and on two such ID site per visit on days 0, 3, 7 and 28. Day 0 is the day of first dose administration of ID RV and may not be the day of animal bite.

Our study highlights the economic advantages of using ID regimen, as theoretically only 0.8 ml of vaccine is needed for each patient resulting in use of less than 1 vial/patient as opposed to 5 vials/patient that receive PEP using IM route.(2) In case of PVRV which is used in our preventive clinic, about 10 lakhs of rupees for 2006, 2007 and 20 lakhs for 2008 could be saved per year if ID route of administration had been followed in our clinic as shown in Table 1. In this regimen, only four visits are needed to complete vaccination. Day 14 is skipped here as compared to IM regimen. So by this, we are able to reduce the indirect cost involved in terms of man hour cost, travel time and expenses for that visit. Vaccine shortage is a problem in our clinic and also in most Government hospitals and most of those who turn up for treatment cannot afford to buy the complete schedule of vaccines as each dose ranging from Rs.280 to Rs.300. Data from preventive clinic shows that patients attending the clinic and ARV used is increasing year by year, adding financial burden for the purchase of ARVs. To address these issues, where vaccine and money are in short supply, ID route is ideal in terms of economic benefits, safety and efficacy. This reduces the cost of vaccination by about 70-80% and this clearly makes an attractive option for resource-starved countries like ours.(3) With commitment and effort, an ideal IDRV vaccine clinic can be set-up. ID administration requires some amount of technical skills which may be imparted by training health inspectors and staff nurses.

**Table 1:** Comparison between IM and ID regimen in terms of cost incurred with PVRV

Vaccines	Route	2006	2007	2008	Full course of vaccination*
Purified vero cell vaccine Rs.280/vial	IM	Rs.15,58,480/-	Rs.16,00,760/-	Rs.29,26,280/-	Rs.1,400/-
	ID	Rs.4,99,800/-	Rs.5,12,400/-	Rs.9,36,600/-	Rs.450/-
Projected percentage (%) reduction in cost with ID regimen		67.98	67.99	67.98	67.86

* Full course of vaccination: IM regimen – 5 visits, 5 vials: ID regimen – 0.2ml × 4 visits (0.8ml) (<1ml)
